# Enhancing behavioral change with motivational interviewing: a case study in a Cardiac Rehabilitation Unit

**DOI:** 10.3389/fpsyg.2015.00298

**Published:** 2015-03-18

**Authors:** Giada Pietrabissa, Martina Ceccarini, Maria Borrello, Gian Mauro Manzoni, Annamaria Titon, Ferruccio Nibbio, Mariella Montano, Gianandrea Bertone, Luca Gondoni, Gianluca Castelnuovo

**Affiliations:** ^1^Psychology Research Laboratory, Istituto Auxologico Italiano IRCCS, Saint Joseph Hospital, VerbaniaItaly; ^2^Department of Psychology, Catholic University of Milan, MilanItaly; ^3^Faculty of Psychology, University of Bergamo, BergamoItaly; ^4^Cardiac Rehabilitation Unit, Istituto Auxologico Italiano IRCCS, Saint Joseph Hospital, VerbaniaItaly

**Keywords:** heart failure, cardiovascular disease, cardiac rehabilitation, motivational interviewing, Brief Strategic Therapy, behavioral change, adherence, self-care

## Abstract

**Background:** Psychological interventions in cardiac rehabilitation programs appear relevant in as much they significantly contribute to achieve the goals of rehabilitation, to reduce the risk of relapses and to improve patients’ adherence to therapy. To this aim, motivational interviewing (MI) has shown promising results in improving motivation to change and individuals’ confidence in their ability to do so.

**Objective:** The purpose of this article is to integrate theory with practice by describing a three-session case scenario. It illustrates how MI’s skills and strategies can be used to enhance heart-healthy habits. MI may be synergistic with other treatment approaches and it is used here in conjunction with brief strategic therapy.

**Conclusion:** By the use of MI principles and techniques, the patient reported an increase in his motivation and ability to change, developing a post discharge plan that incorporates self-care behaviors.

**Clinical Implications:** MI may be effective in motivating and facilitating health behavior change among obese patients suffering from heart failure.

## Introduction

In Europe, over four million people die from disease of the heart and circulatory system (CVD), which constitutes the main cause of death among adults and elderly. The most common forms of CVD are coronary heart disease (CHD) and stroke (Nichols et al., 2012). The favorable position of Italy in terms of CVD has been consistently documented in several epidemiological studies (Menotti, 1989; Keys, 1997; Giampaoli et al., 2006). However, CHD still represents one of the principal causes of death in Italy, accounting for 13% of general mortality and 32% of circulatory system deaths in the country (Palmieri et al., 2010).

There are many risk factors associated with CVD. Some of them, such as family history, ethnicity and age, cannot be changed. Other risk factors that can be treated or changed include tobacco exposure, high blood pressure (hypertension), high cholesterol, obesity, physical inactivity, diabetes, unhealthy diet, and harmful use of alcohol (Laufs and Bohm, 2000; Day and Goldlust, 2010; Capodaglio et al., 2013).

Over recent decades, industrialized countries have shown a slight but steady decline in the occurrence of certain cardiovascular risk factors (Donfrancesco et al., 2008). Specifically, the improvement in treatment medications has led to a reduction of both systolic blood pressure mean values and hypertension (Vanuzzo et al., 2004; Palmieri et al., 2010). However, social, economic, cultural, political, and environmental factors currently promote and support the adoption of unhealthy behaviors (Molinari and Compare, 2006). Lack of physical activity, smoking status and unhealthy diet still represent a challenge, contributing to the development of major cardiovascular risk factors (obesity and elevated blood pressure, blood sugar, and blood cholesterol; Rozanski et al., 1999). Therefore, the role of overweight and obesity in increasing the risk of CVD becomes clear. In particular, obesity, which is defined as a BMI higher than 30, represents, especially among women, an independent predictor of CVD (Hubert et al., 1983; Pietrabissa et al., 2012).

It also represents a major risk factor for hypertension, hypercholesterolemia, insulin resistance, type 2 diabetes, glucose intolerance, left ventricular hypertrophy, and functional capacity (FC; Proietti et al., 2012; Sadeghi et al., 2013). Currently, 80% of patients referring to CR programs are overweight or obese (Gunstad et al., 2007). Recent studies have also provided epidemiological evidence that psychosocial attributes, such as depression, anxiety, personality traits, social isolation, and chronic stress (Krantz, 1980; Ounpuu et al., 2001; Rosengren et al., 2004; Cipolletta et al., 2014; Curhan et al., 2014), may act as mediators, and even directly as causal factors, for CVD (Manzoni et al., 2011a; Neylon et al., 2013) among healthy populations (Hemingway et al., 2001; Kuper et al., 2002). These may influence health related behaviors (Rozanski et al., 1999) and constitute barriers to both individuals’ recognition of the need for medical care and adherence to treatment, thus determining a more rapid progression of the disease (Brunner, 1997).

Whether psychotherapeutic and psychopharmacological interventions could prevent CVD or alter mortality in patients suffering from this chronic condition still remain to be clarified (Spatola et al., 2014). Contrarily, factors that increase the risk of CVD are globally recognized, and public health approaches to prevention should seriously consider them. Poor diet, physical inactivity and smoking habits remain especially important behaviors to address (Ignarro et al., 2007). However, helping patients to change unhealthy behaviors undoubtedly represents one of the most challenging tasks faced by healthcare providers (Burke et al., 1997; Miller et al., 1997; Roter et al., 1998; Brodie et al., 2008). A post visit research revealed 30–60% of medical information discussed in an encounter being forgotten, and 50% of treatment regimens not followed to their fullest extent (Heynes et al., 1981).

Enhancing motivation and confidence in patients’ self-management skills (Narayan et al., 2011) can increase a persons’ quality of life (Manzoni et al., 2010), which represents an important predictor of greater adherence to treatment regimens over time (Elley et al., 2003; Brodie et al., 2008; Manzoni et al., 2011b; Castelnuovo et al., 2014). Effective interpersonal communication skills of health providers are essential techniques that can be used to accomplish this task. Working together with the patient, the professional must offer gentle guidance to motivate individuals to move toward optimal health, and it is responsibility of the provider to understand how to arrange the conversation and which communication strategy is best to use according to the patients’ own values and interests. As stated by Donald deAvila Jackson “*Impossible patients do not exist, there are just unable therapists.*” In fact, non-effective encounters often result in barriers to optimal self-care. Although patients can seem unmotivated to change, everybody has goals and aspirations (Stawnychy and Riegel, 2013). However, since the perceived costs of the dysfunctional habit may not yet outweigh its benefits, frequently people show ambivalence about whether the problematic behavior really needs to be changed (Pietrabissa et al., 2012) and the way in which practitioners listen and talk with them substantially influence their personal motivation for behavioral change (Rollnick et al., 2008). A study shows that, during office visits, clinicians would interrupt patients disclosures during office visits after only 18 s, and this despite the clients’ ability to identifying issues that they would like to address (Beckman and Frankel, 1984). Assessing and working on patients’ motivation to change seems, therefore, crucial for successful rehabilitation programs (Pietrabissa et al., 2013) and for this reason several motivational approaches have been included as adjuncts into other therapeutic protocols (Pietrabissa et al., 2012; Waller et al., 2012). One promising method is MI, which can be defined as a person-centered form of guiding counseling designed to strengthen a persons’ own commitment to change (Miller and Rollnick, 2013). This directive method enhances individuals’ intrinsic motivation by encouraging them to consider their current behavior and to explore this in relation to their own interests, values and aspirations (Brodie et al., 2008). Assisting individuals to work through their ambivalence about behavioral change, MI appears to be particularly effective for those who are initially resistant to change (Rollnick and Miller, 1995; Butler et al., 1999; Resnicow et al., 2001). It has been found effective in fostering change across a wide range of conditions and settings (Burke et al., 2003; Rubak et al., 2005; Miller and Rose, 2009) and it has been applied to many chronic disease behaviors (Colby et al., 1998; Dunn et al., 2001; Van Nes and Sawatzky, 2010). MI counselors rarely attempt to convince or persuade clients, but use a non-judgmental and encouraging climate making them feel comfortable, then verbally expressing both the positive and negative aspects of their current, dysfunctional behavior (Shinitzky and Kub, 2001). Critical component of MI are specific techniques and strategies such as *reflective listening* and *open ended-questions* which, while reinforcing the therapeutic bond, allow patients to define goals in their own words. The technical hypothesis of MI posits that therapist-implemented MI skills are related to clients speech regarding behavior change and that patients speech predicts client outcomes (Resnicow and McMaster, 2012). Thus, *change talk* such as expressed desire, ability, reasons for, and need to change is selectively emphasized. Information is given with patients’ permission (Rollnick and Heather, 1992). Also, self-efficacy and confidence in the ability to change is promoted with the aim to move the person toward a committed plan for change, and to make him/her feel ultimately responsible for the obtained results.

## Case Illustration

This case is one that the primary author experienced during her work as a clinical psychologist in the CR Unit at the Saint Joseph Hospital, IRCCS Istituto Auxologico Italiano, Verbania, Italy. The patient also took part in the Motiv-Heart study, a RCT of BST and MI among cardiac patients joining a CR program. The purpose of the study is to examine whether adding MI strategies to the standard psychological intervention (BST) will result in enhancing the persons’ self-care, leading to long-term improved psychological and medical outcomes. The professional, specialist in BST, completed the training in MI in March 2013, providing the intervention during May of the same year. Treatment fidelity was monitored by a psychiatrist certified in MI (Martino et al., 2008). Sessions were audio recorded and transcribed verbatim to ensure the MI techniques being adherent to the model and to allow the therapist to reflect on and prepare for the next session. Ethical approval of the study was obtained by the Medical Ethics Committee of Saint Joseph Hospital, IRCCS Istituto Auxologico Italiano.

### Problem and Patient Description

The patient, Giorgio (the name has been changed), was admitted to the 1-months CR program with complaints of shortness of breath and everyday life difficulties due to him being significantly overweight. He is a 39 years-old man, resident in the south of Italy. Height 1.66 cms and, at admission to the hospital, weighing 143 kgs. With an initial BMI of 51.9, calculated by dividing the weight (in kg) by the square of the height (in meters), he falls into the highest of the level of obesity (super morbid obesity), presenting increased risk for several health-related conditions. Giorgio was diagnosed with dilated cardiomyopathy, a serious condition in which the heart muscle becomes weakened, therefore unable to satisfy peripheral requirements. He also has a prosthetic heart valve. During the hospitalization, Giorgio underwent an echo to measure his heart’s EF, that is the percentage of blood pumped out of the heart during each heartbeat. His EF was 25%, markedly depressed and further complicated by the presence of life-threatening ventricular arrhythmias. At the 6MWT he achieved a distance of 100 m which represents 20% of the predicted value. Several studies have reported that the 6MWT is a reliable measure of increased mortality among cardiac patients, with the distance of less than 300 m being a strong indicator of poor prognosis (Daullxhiu et al., 2011; Zieliñska et al., 2013). The results of 24-h Holter monitoring also revealed ventricular extrasystoles and the sleep study confirmed the presence of sleep apneas**,** a disorder characterized by pauses in breathing during sleep. The relation between sleep apneas and heart failure is two-way in many cases, further increasing the risk of medical complications. On admission to hospital, the mean of the first 3 days measuring of systolic blood pressure was 108. The Harris–Benedict equation estimates an individual’s resting energy expenditure (REE) of 2589 kcals, and 1800 daily kcals were considered necessary for guaranteeing weight loss by a professional dietician.

Giorgio has had three previous admissions to this cardiac unit, in 2007, 2008, and 2012, respectively. His weight at last discharge was 122.3 kg, following which he affirmed to have successfully maintained a healthy lifestyle, albeit for a short time. A few months after discharge he started to progressively spend more time at home, abandoning both the commitments of the gym and the diet. He never stopped smoking. Giorgio is married and has three children aged 6, 12, and 17. While his wife spends all day at work, Giorgio, due to his complex medical condition, is currently unemployed and looks after their children in a full time capacity.

This case presents a number of common themes in the management of care for challenged heart patients. Giorgio had multiple readmissions for heart failure, each time getting more frustrated and discouraged with himself. He could verbalize proper self-care behaviors and yet was unable to prevent further relapses. According to MI, this incongruity can be explained as *ambivalence,* which represents a normal part of the process of change (Rollnick and Heather, 1992). Ambivalence is simultaneously wanting and not wanting something, or wanting both of two incompatible things. Giorgio is perfectly aware that he ought to quit smoking, exercise regularly and stay on the diet, but other motives conflict with doing the right thing (Miller and Rollnick, 2013). Believing that Giorgio was not determined or compliant to succeed, healthcare providers emphasized the negative aspects of his poor self-care behavior, explained to him why change is important and advised the patient about the necessity to comply with their advice once again.

However, he has already heard the “good” arguments for changing, not only from others but also from a voice within. Moreover, the reward that Giorgio derives from not changing his problematic life-style have not been taken into account. The role of effective healthcare providers must then include an understanding of the interpersonal skills that can be used to allow patients to discover their own reasons for change and to motivate them moving toward optimal health.

## Intervention

### First Session

A psychotherapist trained in MI met Giorgio for the first session in the clinic. In 2012, during the previous hospitalization, the patient received psychological support from the same professional. No motivational intervention was provided at that time. After a brief assessment of what happened since the last discharge, the therapist focused on investigating the reasons that had led him to seek re-admission. Assuming a *one-down* position, the practitioner attentively listened to the patient’s story, respecting and valuing him as the expert of his own life. Within the first few minutes, the patient’s perception of lack of control over his health condition emerged. Justified by the fact of taking care of the younger son, he claimed that he had been unable to maintain the diet longer than a month. Also, he had drastically reduced the exits to the point of socially isolating himself. Lack of physical activity and poor diet had led him to progressive weight gain and experiencing significant daily life difficulties. Giorgio was perfectly aware that his behavior was counter-productive for his health status, but he stated he simply could not do otherwise. Reflectively listening, the therapist started focusing on engaging the patient, building interest in change, and eliciting willingness to implement self-care behaviors.

Patient “I have gradually gained weight not realizing it and now... 4 months ago, I have started to think about everything. *And I have to change.”* (Change talk – Need)

Therapist “What has made you feel ill?”

Patient “My children, and especially the youngest one. Two years ago he was 4, and he could not understand what was going on … he did not comprehend that I was not feeling well. But he now begins to realize … he asks me to go out and, understanding my difficulties, he tells me: <Do you feel bad, Dad?>. *It really bothers me*.” (Change talk – Reason)

Therapist “Because you cannot spend all the time that you would like with him. You are worried at being unable to look after him. Young children are full of energy”(Reflective listening).

Rather than focusing on the negative experiences of his failure in following the care plan, the practitioner continued exploring Giorgio’s goals and values. Apart from his own health status, his main motivation for changing were his wife and three children.

Therapist “What really counts for you today? What is important for you now?”

Patient “To get back to a healthier status and spend more time with my children... with my wife too!”

The therapist proceeded clarifying, together with the patient, what to focus on within an ongoing process of change (*agenda mapping* technique). In an attempt to clarify Giorgio’s own perspective on the situation, what he needed, and how to accomplish it, the health care provider addressed the conversation to his children and consort, so as to help him figure out and verbalize the possible connection between his behavior and the adverse consequences.

Therapist “Which behavior, in particular, are you aiming to change?” (Agenda mapping)

Patient “Meanwhile, *I want to lose weight*.” (Change talk – Desire)

Therapist “Perfect! However, losing weight entails both changing diet and enhancing physical activity. Among these, which is your priority?” (Agenda mapping – The focus must be on a single behavior at the time)

Patient “The diet! I believe exercise is too difficult at the moment.”

Therapist “Ok! To change eating habits! Now, I wonder, correct me if I’m wrong... During the previous hospitalizations I suppose you have received proper nutritional education. You have learned how to combine foods and so on …”

Patient “Yes.”

Therapist “Assuming that to maintain a similar diet once discharged is hard, almost impossible I believe.... what have been the greatest difficulties, the biggest challenges you have experienced at home? What has made it hard to change?”

Patient “Initially I was successful! Then I got lost... I do not know why. I have lost 2–3 kgs during the first month …”

Therapist “Very good!” (Support self-efficacy)

Patient “… Then I have stopped to follow the indications because, as I said, I have preferred to cook for my kids than to take care of myself.”

Therapist “So, correct me if I’m wrong, *on one side* you set yourself aside for others, for the sake of your children... that, as you stated, are the most important thing for you. *On the other side*, failing to obtain results in losing weight despite your efforts, have made you upset, and feel like giving up!” (Double-side reflection)

Patient “Yes. Exactly!”

Therapist “You must be frustrated!” (Reflection)

When a person is ambivalent about changing behavior, it is normal to hear two kinds of talk mixed together: *change talk*, the person’s own statements that favor change, and *sustain talk*, the individual’s own reasons for sustaining the status quo. By the use of double-sided reflection the professional captured both sides of the ambivalence. Then, supporting self-efficacy, asking open questions and reflecting back what stated by the patient, the therapist tried to evoke “commitment” language. In fact, when people talk about change themselves, they are more likely to reach their goal than if someone else (such as the clinician, a friend or relative) talks about it (Pascal, 1662). In this way, change talk is self-evocative. Inviting Giorgio to voice his own arguments for health behavior change the therapeutic aim is to move him along the process of change (Rollnick et al., 1993). The encounter proceeded investigating both the advantages and the disadvantages of the problematic situation. Asking for permission, the practitioner also evaluated Giorgio’s point of view about the fact that, when a problem is maintained over time, to some extent it constitutes a secondary gain. The patients showed resistance and avoided answering.

Therapist “What are the benefits you gain behaving in this way? Not to worry about your diet... you told me <I have to cook for my children, they are my priority so I can’t take proper care of the quality of the food for my health needs> ... all combined with lack of physical activity. What advantages are there in behaving this way?”

Patient “What does it mean <the advantages>?”

Therapist “I was wondering, when a person continues to implement behaviors he knows being harmful for himself, for his health... There must be a reason.... Otherwise it’s torturous, a self-destruction, isn’t it? Must be some motives that lead you to act in this way. What do you think?” […]

Patient “Look, I hope to continue following the diet at home … and regarding my previous conduct, looking back … it was just a sort of self-destruction! That’s it!”

Before the end of the first session, the professional assessed Giorgio’s readiness to make a change, as well as how important he felt it was to continue maintaining a healthy lifestyle and the degree of confidence in his ability to do so. On a scale from 1 to 10, he always rated the maximum score. He expressed anger against himself, as well as the clear intention not to seek a new re-hospitalization in the future. The therapist also explored the worst consequences of the patient’s current behavior (*querying extremes* technique).

Summarizing, session one focused on building a therapeutic relationship. After having found Giorgio’s main reasons to change being his wife and children, his ambivalence in modifying unhealthy behaviors was explored and change talk enhanced. Also readiness, importance and confidence about change were assessed.

### Second Session

After a week, the psychotherapist visited Giorgio for the second time. The professional started investigating the course of his daily activities within the hospital, the results of his medical examinations, as well as his state of mind. After a psychiatric consultation he stated that he had to take a pill for his mood, with no beneficial effect. Thus, the expert asked Giorgio what were his thoughts about the matter, positively restructuring the lack of need of the psychopharmacological therapy, then reinforcing his personal resources. The professional also evaluated his understanding of the possibility to discuss the therapy with his cardiologist.

The encounter proceeded exploring any additional worries of the patient. Despite presenting realistic concerns in mind regarding his health status, he expressed sincere need for well-being and clear goals. Regarding his health objectives, he formulated specific plans of action and showed realistic understanding of the time necessary to achieve lasting results *(“I’m hopeful... I give myself a year and a half … I do not believe it would be enough time to get to the point I would like to reach. However, I do hope to get to the point of being 80% independent”).*

Talking more about when and how to change and less about whether and why, the therapeutic purpose became the promotion of Giorgio’s autonomy of decision-making and to further elicit change talk.

Mindful of his significant health difficulties, the patient appeared to be strongly determined to change* (“I do not have the <I can’t do it> in front of me” … “What I would actually like to do, I will do in 2 years, such as ride a bike with my son or going to the beach or take a proper family vacation”).*

While during the previous encounter the patient was asked to think about the worst consequences of his status quo, the therapist carried on inviting Giorgio to imagine the best things that could result from changing, as well as to imagine a changed future (*looking forward* technique).

Giorgio’s thoughts always turned to his family and his concerns were addressed about the fear of not being able to fulfill his role of husband and father anymore. Before asking for admission to the hospital, he stated that it was hard for him to climb a flight of stairs as well as help his wife to go food shopping and, especially, to maintain regular sexual intercourse. He became aware of the level of seriousness of his situation about a month before, and declared himself determined of changing his dysfunctional habits.

Exploring his values also helped identifying Giorgio’s main objective: “*I want to tell you the truth, my ultimate goal, a wish that I have, if I could adjust 80 kgs of weight, I would like to put myself on the heart transplant list*.”

Actively listening and summarizing key points back to the patient, the provider displays genuine interest in what has been said and led Giorgio to reflect about the possibility that his smoking habit could threaten the achievement of his intent.

Patient “... I am currently smoking two cigarettes per day, but for boredom.”

Therapist “When you do not know what to do, in the dead time, you get bored and light a cigarette.”

Patient “I would like to go to the bar (in the hospital) but it implies too much effort for me and I need to stop … or if I can get there I often have to stand because of the lack of seats. Therefore, instead of going to the bar I go to the gazebo, I sit for an hour outside, I smoke a cigarette … then I go back to the room again... that’s what I do.”

The patients seemed to underestimate the importance of quitting smoking, appearing unaware or potentially resistant to change. Despite the strong motivation shown in regulating his eating habits, it looked like smoking for him was an excuse to avoid walking even only for very short distances. Due to the lack of time remaining, the psychotherapist decided to postpone working on Giorgio’s motivation to quit smoking until the next and last session. Thus, she opted for suggesting him some strategic behavioral expedients according to the brief strategic model. On one hand these were aimed at helping the specialist identifying the presence of any resistance in the patient and, whether successfully executed, to break the vicious circle that maintain the problem. Thus, after having asked for permission and verified the patient’s disposition of following the prescriptions, the professional proceeded introducing him the so-called “*Interval technique*”:

Therapist “When you want to smoke a cigarette, wait for half an hour. You are perfectly aware of what is good or bad for your health, and you do not need to hear me say that you should quit smoking. So, when you feel the need for a cigarette, I believe you have two choices: you can simply go, or you can wait for half an hour and, if after having waited for 30 min you still desire to smoke a cigarette, just go! I may be wrong, but I believe that if I tell you not to smoke, as I imagine everybody does, I would make it even more desirable for you … What do you think? Can it be an acceptable option?”

Patient “Ok.”

The therapist then continued with the “*Limits map*”:

Therapist “Similarly, let’s say you want to go from the room to the bar, from the bedroom to the balcony … here you also have two options: you can give up due to your physical impairments, reinforcing your sense of ineffectiveness, or you can try and, day after day, attempt after attempt, measuring your limit. You do not necessarily have to reach the point … just check every day the distance that separates you from the goal, then get back! Next week I’m curious to see where did you get.”

Patient “I must not let my fear of not succeeding get the best of me.”

Therapist “Exactly, you get the point!”

Briefly, while the first technique is essentially aimed at leading the patient to “allow himself to do something to be able to renounce it” so as to “avoid the control that makes losing control,” the latter is directed at bringing Giorgio to focus on the task (in this case to reach a given destination). Doing so, the goal is to distract the patient from his usual “cigarette break” and to make him experience successes progressively reinforcing his self-efficacy at the same time.

### Third Session

Therapist “How was your week? Did you try to put into practice the advice I gave you last time?”

Patient “Not smoking, you mean?”

Therapist “Exactly, were you up for a cigarette?”

Patient “Yes, I was at the bar and I said <now I’m going to have a cigarette> … and then I thought about what you have proposed to me last time … and I said <no! I can wait> ... and I have not smoked in the end.”

Therapist “You have waited for half an hour …”

Patient “I didn’t smoke at all… I didn’t feel like having a cigarette.”

Therapist “Ok, so after half an hour your desire for a cigarette has gone and you decided not to go to smoke.”(Reflection)

Patient “Yes … But this morning I had one cigarette.”

Therapist “Ok, did you wait for 30 min?”

Patient “No, not this time … after the gym, I was going back to the ward but I had time and I said to myself <I will stop here>. Everybody were on the balcony smoking and I said <come on! It’s not that big deal!> ...”

Therapist “Ok … and what about the other trick? The one of ‘touching the limit’?”

Patient “I have done it! … I wanted to go to the bar, I was giving up … but I finally went!””

Therapist “Did you said <I will touch my limit and I will see how far I can get then I will go back”?

Patient “Yes … but I have reached the point!”

Therapist “Perfect! How did you feel?”

Patient “Tired but it was ok.”

Therapist “Great work Giorgio!” (Support self-efficacy)

Patient “Also this morning … I woke up really bored, wanting to do nothing… but I have done another 30 min of physical activity and now I am fine!”

Therapist Very Good! (Support self-efficacy)

Investigating the effects of the prescriptions given to the patient, the professional supported the successes he achieved. In fact, the therapeutic goal was to break the rigid belief of the patient, his sense of inability.

Giorgio experienced his abilities and resources and, according to the strategy logic, this will lead to a gradual change in his way of perceiving and reacting to his reality.

During the previous week, something had changed within the patient’s mind. In fact, for the first time, he felt the need to completely open up to the therapist, revealing the real and intimate reason that has led him to stop taking care of himself. Giorgio had never verbally expressed it before, with an obvious liberating effect. The basic emotion sustaining the problem is the fear, to which an important guilt is linked (“*I’m afraid of getting back to being fit, then eventually relapsing back into the problem*”).

The largest use of reflective listening allowed the therapist to let the patient run the meeting within an evocative process. The professional continued directing the session in a collaborative way, respecting patient’s autonomy as well as his freedom to talk about himself and his problem.

During the encounter, potential barriers to maintaining behavior change were also elucidated, and the MI principles explored during the previous encounters further reinforced.

At the end of the session Giorgio showed large sense of control on hidden difficulties and, by extension, of his life. During the hospitalization he has lost weight and started exercising regularly again, with both physical and psychological benefits.

His weight at discharge was 139,6 (BMI = 50,7) and the measure of his systolic blood pressure was 100.

He made an experience of his abilities, also taking responsibility for the achieved successes. Commitment to change was expressed, by displaying motivation and a plan to alter his behavior within the coming months.

## Discussion

This scenario represents an example of how MI principles and techniques can be strategically and flexibly used in the clinical setting to help patients moving toward the complex continuum of change. Specifically, MI intervention may be helpful for a person suffering from chronic condition at risk for readmission or for a newly diagnosed and overwhelmed patient, as Giorgio was. In fact, although demonstrating knowledge of self-care behaviors, this did not correlate with the action.

By the use of specific communication skills that enlisted patent’s active involvement in the encounter, discrepancies between both the pros and cons of changing and desirable self-care behaviors were acknowledged and explored, in a non-judgmental manner, in service of Giorgio’s own goals. Also, the professional helped to encourage his positive arguments for change by recognizing the patient’s readiness to change and eliciting a plan. It is essential to create a supportive environment where providing individually tailored services, properly considering the patient’s life context and resources.

Over the course of ∼3 h of integrated sessions, Giorgio showed increased sense of control over the disease and greater self-confidence in being able to reach long-term results. He has been able to verbalize the reason that had led him to progressive social isolation and lack of interest in both following the diet and maintaining physical activity, he has also successfully acquired skills to practically manage daily-life difficulties and temptations.

Since the motivation and actions needed to achieve patient’s goals originated from the client himself, and Giorgio took responsibility of his own progress, the chance for successful behavior change has been increased. Too often, in fact, the results achieved during the rehabilitation period are experienced by patients as responsibility of the medical staff, seriously impacting both their medical and psychological outcomes when, once discharged they experience relapses. When performed correctly, MI encourages communication and trust and makes patients active participants in their care (Shinitzky and Kub, 2001; McCambridge and Strang, 2004; Berman et al., 2010; Resnicow and McMaster, 2012; Stenman et al., 2012; Stawnychy and Riegel, 2013; Brand et al., 2013). This intervention represents only one of several factors that may contribute to the patient’s success. Proper medical management, coupled with the use of available resources, are crucial. However, without a willingness to change behavior, no amount of resources will be successful. Through the art of motivating, health care providers can empower individuals to positively influence their level of health.

## Abbreviations

BMI: body mass index
BMR: basal metabolic rate
BST: Brief Strategic Therapy
cms: centimeters
CR: cardiovascular rehabilitation
CVD: cardiovascular disease
echo: echocardiogram
EF: ejection fraction
kcal: Kilocalories
kgs: Kilograms
MI: motivational interviewing
RCT: randomized controlled clinical trial
6MWT: six minute walk test.
